# Mechanisms of toxic smoke inhalation and burn injury: Role of neutral endopeptidase and vascular leakage in mice

**DOI:** 10.1080/15376510902725649

**Published:** 2009-06-30

**Authors:** Sam Jacob, Donald J. Deyo, Robert A. Cox, Daniel L. Traber, David N. Herndon, Hal K. Hawkins

**Affiliations:** 1 Department of Pathology, University of Texas Medical Branch, Galveston, TX, USA; 2 Department of Anesthesiology, University of Texas Medical Branch, Galveston, TX, USA; 3 Shriners Burns Hospital, Galveston, TX, USA

**Keywords:** Neurogenic inflammation, neutral endopeptidase activity, NEP, plasma extravasation, vascular permeability, acute lung injury

## Abstract

The effects of neutral endopeptidase (NEP) in acute inflammation in the lung were studied using a newly developed murine model of smoke and burn (SB) injury. C57BL/6 mice were pretreated with an i.v. dose of a specific NEP antagonist CGS-24592 (10 mg/Kg) 1 h prior to SB injury (n = 5–8/group). Mice were anesthetized with i.p. ketamine/xylazine, intubated, and exposed to cooled cotton smoke (2 × 30 s). After s.c. injection of 1 ml 0.9% saline, each received a 40% total body surface area (TBSA) flame burn. Buprenorphene (2 mg/kg) was given i.p. and resuscitated by saline. Evans Blue dye (EB) was injected i.v. 15 min before sacrifice. Lung wet/dry weight ratio was measured. After vascular perfusion, lungs were analyzed for their levels of EB dye and myeloperoxidase (MPO). In mice pretreated with CGS-24592 followed by SB injury the EB levels were significantly higher (61%, *p* = 0.043) than those with SB injury alone. There was a significant increase (144%, *p* = 0.035) in EB dye in animals with SB injury alone as compared to shams. In mice pretreated with CGS-24592 prior to SB injury wet/dry weight ratios were significantly (27%, *p* = 0.042) higher compared to animals with SB injury alone. CGS-24592 pretreatment also caused a significant increase in MPO (29%, *p* = 0.026) as compared to mice with SB injury alone. In conclusion the current study indicates that specific NEP inhibitor CGS 24592 exacerbates the SB-induced lung injury and inflammation in mice.

## Introduction

Neurogenic inflammation is regarded as a first line of defense and protects tissue integrity when noxious conditions threaten normal body functions (Thorning et al 1982; Richardson and Vasko 2002; Birklein and Schmelz 2008). However, severe or prolonged noxious stimulation may result in the inflammatory response mediating injury rather than facilitating repair. Airway neurogenic inflammation is caused by neuropeptides released from peripheral nerve endings of sensory neurons within the airways, and is characterized by plasma protein extravasation, airway smooth muscle contraction, and elevated mucus secretion (Nadel 1991; Barnes 1996). In vivo the concentration of released neuropeptides depends on both release and degradation of enzymes, and Neutral endopeptidase (NEP) is the principal degradative enzyme. NEP cleaves peptide bonds on the amino side of hydrophobic amino acid residues, leading to inactive fragments. NEP is present in lungs, brain, intestine, and bone marrow (Roques et al. 1993).

In the lung, NEP is abundantly expressed in epithelial cells (Johnson et al. 1985). Inhibition of NEP by thiorphan or phosphoramidon leads to increased secretion of NEP (Edwards et al. 1999). In several cases NEP is secreted by cells that have tachykinin receptors. The key role of NEP in limiting and regulating the neurogenic inflammation provoked by different stimuli has been demonstrated in multiple recent studies (Nadel 1991; Maria et al. 1998; Chung 2006). It has been shown that a variety of factors that stimulate clinically relevant airway inflammation, including viral infections, allergen exposure, inhalation of cigarette smoke, and other respiratory irritants, also reduce NEP activity, thus enhancing the effects of neuropeptides within the airways. The degradative activity of NEP is not limited to neuropeptides, but also affects other significant peptides including angiotensin II and brakykinin (Katayama et al. 1991; Turner et al 1993; Yang et al 1997).

Recent studies from this laboratory have demonstrated increased vascular permeability in a mouse model of combined SB injury (Jacob et al. 2008). There was a significant (*p* < 0.05) increase in lung Evans Blue dye content, wet/dry weight ratio, and MPO content 24 and 48 h after SB injury in Balb/C and C57BL/6 mice, respectively, indicating increased plasma extravasation and edema.

On the basis of these observations, reduction of NEP activity may be regarded as part of a process that alters neurogenic airway responses from their physiological and protective function to a detrimental role that increases and perpetuates airway inflammation (Figure 4). Better knowledge of the regulation of the neurogenic pathway of airway inflammation would provide valuable information on the mechanism of acute lung injury. We hypothesized that inhibition of NEP would exacerbate SB injury, leading to more plasma extravasation and more severe airway inflammation. To test this, we studied whether blocking NEP with a specific inhibitor, CGS 24592, would increase vascular leakage and edema and inflammation.

## Materials and methods

### Animal preparation and treatment

Male C57BL/6 mice weighing 19–21 g (5–6 weeks old) were obtained from Charles River Laboratories (Wilmington, MA). Mice were acclimatized to the animal facility for at least 1 week before the experiments were started. All procedures have been reviewed and approved by the Animal Care and Use Committee of the University of Texas Medical Branch specifically for use in these research studies.

### Tracheal intubation and cotton smoke exposure

Briefly, mice were anesthetized with i.p. injection of ketamine (50 mg/kg), and xylazine (10 mg/kg). Once adequately anesthetized, mice were suspended by their upper incisors on a custom made intubation stand that allows direct visualization of the mouse glottis. A 20 gauge plastic cannula with a Y-shaped adaptor was constructed from readily available parts and inserted into the trachea using a specially constructed laryngoscope made from an aluminum ‘mag light’ (Mag Instrument Inc., Ontario, CA) flashlight fitted with a fiber optic illuminator, under direct vision using a head-mounted jeweler's magnifier.

A custom-made miniature smoke generator was arranged so that smoldering cotton toweling could be kept burning by a regulated flow of air. Smoke emerging from a cooling coil was delivered through plastic tubing to the Y-connector attached to the endotracheal tube at near atmospheric pressure. A pressurized air source was attached to the smoke generation chamber to control the flow and density of smoke delivered to the Y-tube. Mice were allowed to breathe normally during smoke exposure. Mice were exposed to two 30-s intervals of smoke inhalation, breathing room air between exposures, followed by 48 h of reaction and recovery.

### Burn injury

After smoke exposure, the back and bilateral flanks of each mouse were shaved, and 0.9 ml of normal saline was injected subcutaneously along the vertebral column. This subcutaneous liquid prevents thermal injury to internal organs such as the spinal cord and liver (Stephenson et al. 1975). A Zetex cloth with a square window was placed over each mouse during the burning procedure to prevent thermal injury to other structures and to confine the burn area. Each mouse received a brief (less than 10 s) exposure to a Bunsen burner flame. The flame was applied just long enough to induce retraction of the skin. The Zetex window allows for administration of a 40% total body surface area, full thickness burn.

Mice were resuscitated with normal saline (4 ml/kg/% TBSA burn, i.p.). The endotracheal tube was removed as the mice recovered. Animals were given a daily dose of buprenorphene (0.1 mg/kg, i.p.) starting immediately after the injury for post-procedure analgesia. At the end of the experimental time period, mice were killed by isoflurane overdose followed by cervical dislocation. Histologic study demonstrated full thickness dermal injury and showed no signs of necrosis or other recognizable changes in the portion of the liver under the site of injury. Sham control animals were anesthetized and intubated in the same manner as experimental animals, but were allowed to breathe room air rather than given smoke exposure. They were also shaved and injected along their spines with saline, but not burned. The sham control mice received the same volume of saline resuscitation and the same routine analgesia as the animals injured by burn and smoke inhalation. In addition, mice were studied for comparison with the sham animals that were not intubated or shaved or injected with saline (‘untouched’ control animals).

### Preparation and administration of drug

The specific NEP inhibitor (CGS 24592; (S)-*N*-[2-(phosphonomethylamino)-3-(4-biphenylyl)-propionyl]-3-aminopropionic acid) was kindly provided by Novartis (Cambridge, MA). CGS 24592 was prepared at 1 mg/mL in sodium bicarbonate buffer and sterilized through a 0.22-μm filter and kept frozen until use. The drug was dissolved in HPLC grade H_2_O at the concentration of 10^−3^ M, filtered through a 0.22-μm filter (Millipore), and stored at −70°C. Mice were treated i.v. at a dose of 10 mg/kg 1 h prior to SB injury and then every 24 h over the 48-h period.

### Tissue sampling

Animals were deeply anesthetized at 48 h after SB injury, the chest of each mouse was opened, and a median sternotomy was performed. The lungs were excised in one block and the left and right lungs were separated. Both lungs were snap-frozen in liquid nitrogen for determination of wet/dry weight ratio.

### Evans Blue extravasation

Sham and SB injured mice were injected with Evans Blue dye (EB, 20 mg/kg) via the tail vein 30 min before the termination of the experiment to assess vascular leakage (Wang et al. 2002). After perfusion of the lungs with PBS as described previously (Jacob et al. 2008), the lungs were excised *en bloc* and dried on filter paper. After weighing, the lung tissue was snap frozen in liquid nitrogen and stored at −80°C. At the time of assay, lung tissue was manually homogenized using a bead beater (Biospec Products, Bartlesville, OK) and incubated with two volumes of formamide (Fisher Scientific, Fair Lawn, NJ) for 18 h at 60°C. The supernatant was collected after centrifugation at 5000 g for 30 min. The optical density of the supernatant was determined spectrophotometrically at a wavelength of 620 nm. Levels of EB in samples were quantitated using a standard curve prepared with known amounts of EB, and the data are expressed as nanograms EB/mg lung.

### Wet-to-dry-weight ratio

Lungs were removed and placed in preweighed glass vials, then dried for 48 h in an oven at 65°C and reweighed, using a balance capable of indicating the weight to the nearest 0.1 mg, to determine the ratio of wet-to-dry weight, a measure of total tissue water content (Zhao et al. 2006).

### Determination of myeloperoxidase

Sequestration of polymorphonuclear neutrophils (PMNs) indicated by MPO activity was measured in lung tissue homogenates using an MPO-EIA kit (Oxis Inc, Portland, OR), following the manufacturer's protocol. Briefly, lungs were perfused with PBS as described earlier and homogenized in TBS-T buffer using a bead beater and centrifuged at 13,000 g for 20 min. The protein content of the supernatant was assessed using the Bradford assay (Bradford 1976). To assess MPO, 100 μl of the supernatant was added to the micro wells in triplicate and incubated for 1 h at room temperature. Plates were washed using washing buffer and diluted anti-MPO was added to each well. After 30 min incubation, plates were washed and avidin alkaline phosphatase was added followed by *p*-nitrophenol phosphate (pNPP) solution. The reaction was allowed to develop a pale yellow color for 30 min and was then terminated by adding 50 μl stop solution, and absorbance was immediately read at 405 nm using a TECAN-GENios microplate reader (Durham, NC). Myeloperoxidase activity was quantitated using a standard curve with known amounts of MPO, taking care that samples were compared to the linear portion of the standard curve.

### Statistical analysis

Values are shown as the mean ± SE of at least five determinations (n = 5). Statistical significance of the results was conducted by one-way analysis of variance followed by Dunnette's post-hoc test. The level of significance in all experiments was defined as *p* < 0.05.

## Results

### Plasma extravasation in mouse lung

**Figure 1:**
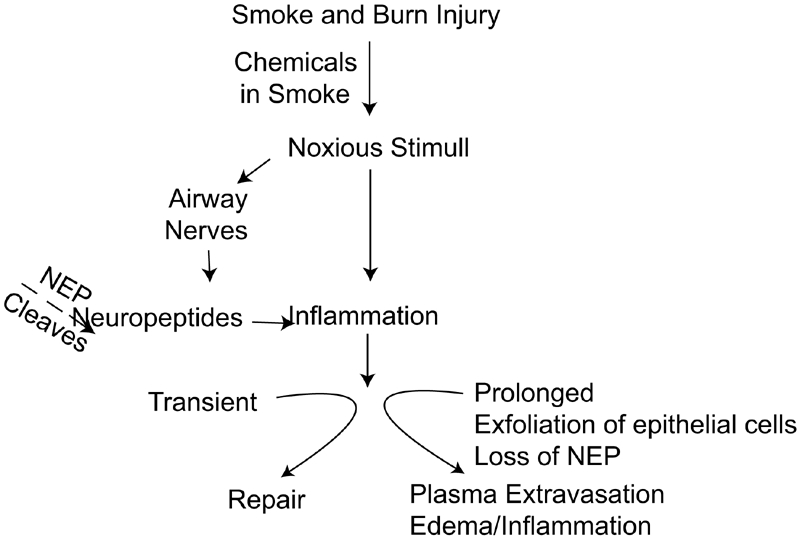
Proposed pathway for the role of NEP in lung injury following SB injury in mice. Initial neurogenic inflammation induced by SB induced noxious stimuli will be prolonged and intensified due to decrease or lack of NEP, causing excessive plasma leakage, edema and neutrophil infiltration.

Levels of EB were quantitated in mice pretreated with specific NEP antagonist CGS 24592 followed by SB injury. The data indicate significantly higher (62%, *p* = 0.043) levels of EB in NEP antagonist pretreated and SB injured mice as compared to untreated animals with SB injury at 48 h after injury (Figure 1). As expected, Evans Blue dye levels were also significantly (144%, *p* = 0.036) higher in SB injured mice as compared to respective sham controls. Pretreatment with CGS 24592 alone caused a significant increase (69%, *p* = 0.016) in EB levels as compared to respective sham animals pretreated with bicarbonate buffer.

### Lung wet/dry ratio

**Figure 2:**
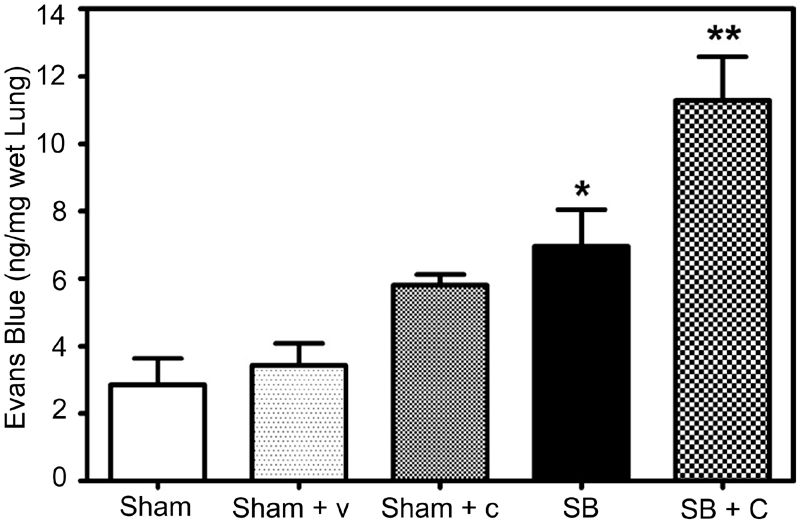
Effect of NEP antagonist pretreatment in plasma extravasation: A significant increase (**p = 0.0432) in EB levels was observed in NEP antagonist pretreated mice (Checked Bar, SB+C) 48 hr following SB injury compared to animals with SB injury alone (SB, black filled bar). EB levels were also significantly (*p =0.0356) increased 48 hr following SB injury alone (black filled bar) compared to respective sham animals (open bar). Pretreatment with CGS 24592 alone (Sham + C, speckled bar) caused significant increase (69%, p = 0.016) in EB levels as compared to respective sham animals pretreated only with the drug vehicle, bicarbonate buffer (Sham + V, dotted bar). EB levels were quantitated from an authentic standard curve with known concentrations of EB. Values are means ±SE of at least 5 mice/group.

Wet/dry weight ratio was calculated as described earlier. Wet–dry weight ratios were significantly elevated (27%, 0.043) in mice pretreated with CGS-24592 followed by SB injury as compared to corresponding SB control animals (Figure 2). The data also indicate that at 48 h following smoke and burn injury there was a significant increase (57%, 0.022) as compared to sham animals. Pretreatment with CGS 24592 alone (Sham + C, speckled bar) caused a slight decrease in wet/dry weight ratios as compared to respective sham animals pretreated with bicarbonate buffer. Sham animals showed an insignificant increase (32%, *p* = 0.137) in wet/dry weight ratio as compared to untouched control animals (data not shown).

### Myeloperoxidase activity

**Figure 3:**
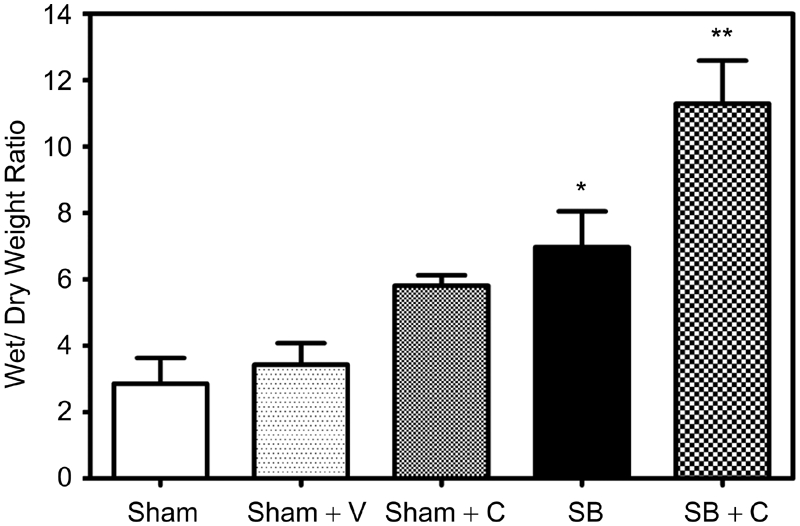
Effect of NEP antagonist pretreatment on edema: A significant increase (** p = 0.0425) in wet/dry weight ratios was indicated in NEP antagonist pretreated mice (Checked Bar, SB+C) 48 hr following SB injury compared to animals with SB injury alone. (SB, black filled bar). Wet/dry weight ratios were also significantly increased (*p = 0.0218) 48 hr following SB injury alone (black filled bar) compared to respective sham animals (open bar). Pretreatment with CGS 24592 alone (Sham + C, speckled bar) caused a slight decrease n wet/dry weight ratios as compared to respective sham animals pretreated with bicarbonate buffer (Sham + V, dotted bar). Values are means ±SE of at least 6 mice/group.

Levels of MPO were determined using a sandwich ELISA kit as described, following thorough perfusion of the pulmonary arterial system with normal saline. Because the lungs underwent vascular perfusion prior to analysis, the determination of MPO includes neutrophils that are firmly adherent to pulmonary endothelium as well as those that have extravasated from vessels. In mice pretreated with CGS24592 followed by SB injury there was a significant increase (29%, *p* = 0.026) in MPO levels as compared to untreated mice with SB injury alone (Figure 3). As expected there was a significant increase in MPO levels in SB injured animals compared to respective sham animals (142%, *p* = 0.0056). Pretreatmen twith CGS 24592 alone (Sham + C, speckled bar) caused a slight insignificant increase in MPO activity as compared to respective sham animals pretreated with bicarbonate buffer.

**Figure 4:**
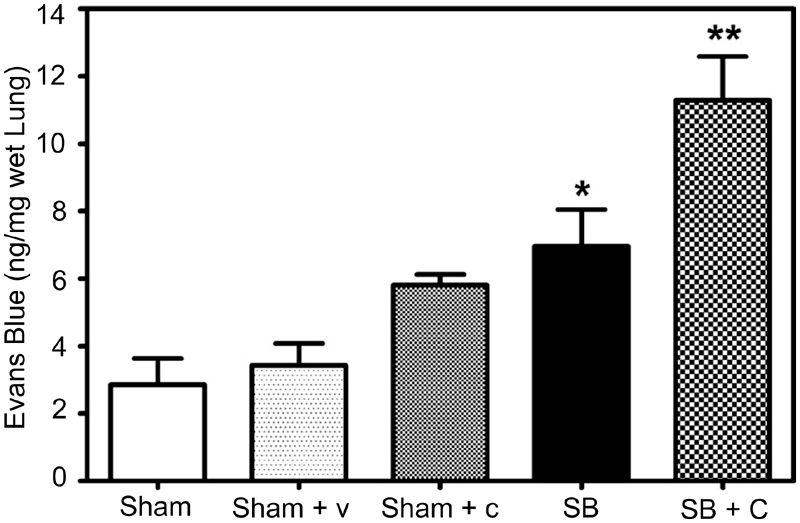
Effect of NEP antagonist pretreatment on neutrophil infiltration and inflammation: A significant increase (**p = 0.0255) in MPO levels was observed in NEP antagonist pretreated mice (Checked Bar, SB+C) 48 hr following SB injury compared to animals with SB injury alone (SB, black filled). MPO levels were also significantly increased (*p =0.0045) 48 hr following SB injury alone (black filled bar) compared to respective sham animals (open bars). MPO levels were quantitated from an authentic standard curve with known amounts of MPO. Pretreatment with CGS 24592 alone (Sham + C, speckled bar) caused a slight insignificant increase in MPO activity as compared to respective sham animals pretreated with bicarbonate buffer (Sham + V, dotted bar). Values are means ±SE of at least 5 mice/group.

## Discussion

Following burn trauma, toxic smoke inhalation has been recognized as a major cause of mortality (Crapo 1981). Previous reports from this and other laboratories have indicated that toxic smoke causes progressive injury to the airways, followed by injury to lung parenchyma (Barrow et al. 1990; Dost 1991; Traber and Herndon 1991; Bidani et al. 1999). Even though rapid release of chemical mediators in the airways is a suggested mechanism for injury, there is limited understanding of the underlying processes that lead to delayed parenchymal injury. Neurogenic inflammation is regarded as a first line of defense and protects tissues when noxious conditions threaten normal body functions. However, severe or prolonged noxious stimulation may result in the inflammatory response mediating injury rather than facilitating repair (Day et al. 2005).

Plasma extravasation and edema are secondary responses to neurogenic inflammation caused by release of substances such as substance P, calcitonin gene-related peptide CGRP, and other neuropeptides from primary sensory nerve terminals (Solvay and Leff 1991; Nadel 1991; Richardson and Vasko 2002). The local concentration of released neuropeptides depends on both release and degradation, and the principal degradative enzyme is NEP, which is abundantly expressed in epithelial cells of the lung (Johnson et al. 1985). Multiple factors that stimulate clinically relevant airway inflammation, including viral infections, allergen exposure, inhalation of cigarette smoke, and other respiratory irritants, also reduce NEP activity, thus enhancing the effects of neuropeptides within the airways. Previous studies have indicated the proinflammatory effects of NEP antagonists including the specific NEP inhibitor CGS 24592 (Damas et al. 1996; Pham et al. 2000; Nicolau et al. 2003). These agents caused plasma extravasation and vascular permeability in various animal models.

The current study was undertaken to examine whether inhibition of NEP would exacerbate SB injury leading to more plasma extravasation and more severe airway inflammation. To test this, we studied whether blocking NEP with a specific inhibitor, CGS 24592, would increase vascular leakage, edema, and inflammation.

The current study indicates that NEP inhibition by CGS 24592 caused a significant increase (*p*<0.05) in plasma extravasation and wet/dry weight ratios 48 h after SB injury as compared to animals with SB injury alone (Figures 2 and 3). Sham animals showed an insignificant increase (32%, *p* = 0.137) in wet/dry weight ratio as compared to untouched control animals (data not shown). Unexpectedly, it was found that NEP antagonist pretreatment also led to increased plasma extrasation in uninjured sham animals. It is possible that neurogenic inflammation may arise during the process of intubation, as wet/dry weight ratios also increased in sham animals. Previous studies have indicated increased MPO activity following NEP inhibition (Umeno et al. 1989; Barbara et al. 2003). The current study further substantiates that inhibition of NEP exacerbates SB injury as there was a significant increase in MPO activity in CGS 24592 pretreated animals with SB injury compared to those with SB injury alone (Figure 3).

The results of the study can be explained based on the proposed pathway summarized in Figure 1. Chemicals in smoke and burn injury stimulate airways towards relatively transient neurologic inflammation. When this noxious stimulus is prolonged, more epithelial cells will be lost, leading to further loss of NEP, plasma extravasation, edema, and inflammation. Thus the current findings emphasize the importance of NEP in the pathogenesis of airway and lung injury following combined burn and smoke inhalation injury in mice. It is well known that one of the earliest reactions to inhalation of cooled cotton smoke in the sheep is detachment of ciliated epithelial cells in the trachea. By 48 h after smoke inhalation, almost all columnar cells in the trachea and bronchi have been lost (Abdi et al. 1990). Since columnar epithelial cells are the principal source of neutral endopeptidase in the airways, detachment of columnar cells may lead to loss of much of the NEP activity after smoke inhalation injury.

An alternate pathway is based on previous reports from this and other laboratories that indicated a delayed lung injury following smoke inhalation (Traber and Herndon 1991; Speyer et al. 2004). Inflammatory mediators are thought to increase the release of other chemicals that may contribute to tissue injury in different stages of smoke inhalation injury. They observed intense acute inflammation in the proximal trachea 3 h after injury, and it took many more hours to extend the injury to the small peripheral airways. The other striking feature of this reaction is that it persists for a long time, becoming more severe and affecting a longer segment of the trachea over the course of at least 48 h. Therefore, the delayed lung injury may not follow a similar pathway compared to that incited by the initial noxious stimuli described in Figure 1. Instead there may be other mechanisms which continue to stimulate neutrophil emigration long after the initial injury, some sort of positive feedback loop may be involved, and/or the acute inflammatory reaction is not normally terminated.

The most likely sequence of events in the initial induction of inflammation is that the chemicals in smoke stimulate impulses in sensory and autonomic nerves, which release substance P and calcitonin-gene-related peptide (CGRP) near the lining epithelium (Lee and Widdicombe 2001). Concurrent and progressive loss of NEP activity might be expected to increase the intensity and duration of the pro-inflammatory effects of neuropeptides in the airways. In conclusion, our study indicates that inhibition of NEP by CGS 24592 would exacerbate SB injury, leading to more plasma extravasation and severe airway inflammation. Further research using large animal models is needed to investigate the exfoliation of epithelium and the release of CGRP near the lining epithelium as a potential mechanism in the pathogenesis of burn and smoke inhalation injury.
